# Association between blood cadmium and depression varies by age and smoking status in US adult women: a cross-sectional study from NHANES 2005–2016

**DOI:** 10.1265/ehpm.24-00050

**Published:** 2024-06-22

**Authors:** Yewei Ji, Jinmin Wang

**Affiliations:** Department of Internal Neurology, The Second People’s Hospital Affiliated to Fujian University of Traditional Chinese Medicine, Fuzhou 350003, China

**Keywords:** Blood cadmium, Metal exposure, Depression, NHANES, Cross-sectional study

## Abstract

**Background:**

Cadmium, a toxic metal, is widely encountered in diverse environmental contexts. Despite its pervasive exposure, there is limited research on the association between blood cadmium levels and depression, especially among females. This study aimed to investigate the relationship between blood cadmium levels and depression in adult women.

**Methods:**

Data spanning 2005–2016 from the National Health and Nutrition Examination Survey (NHANES) were selected. Depression was diagnosed with the Patient Health Questionnaire (PHQ-9, score ≥10). Multiple logistic regression, multiple linear regression, and smoothed curve fitting were used to investigate the relationship between blood cadmium and depression. Subgroup analyses and interaction tests were performed to evaluate the stability of this association across populations.

**Results:**

A total of 1,173 individuals were diagnosed with depression. The heightened prevalence of depression was linked to increased blood cadmium levels, a trend that persisted even after quartering blood cadmium. In the fully adjusted model, each incremental unit of blood cadmium was associated with a 33% rise in the prevalence of depression (OR = 1.33, 95% CI: 1.21–1.45). Participants in the highest quartile were 63% more likely to experience depression compared to those in the lowest quartile of blood cadmium (OR = 1.63, 95% CI: 1.15–2.30), and PHQ-9 score increased by 0.73 (β = 0.73, 95% CI: 0.30–1.17). This positive association may be relevant to the general population.

**Conclusions:**

Blood cadmium levels are associated with depression in adult women, and this association varies by age and smoking status.

**Supplementary information:**

The online version contains supplementary material available at https://doi.org/10.1265/ehpm.24-00050.

## 1. Introduction

Depression, a prevalent mental illness characterized by feelings of sadness and hopelessness, poses a significant threat to human health amidst intense social competition and the accelerated pace of modern life [1]. The surge in psychological and physiological stress levels elevates the risk of affective psychiatric disorders, potentially impacting the quality of life and social stability. Globally, approximately 350 million people, constituting 4.4% of the world’s population, are currently grappling with depression [2]. While the etiology of depression remains incompletely understood, a growing recognition of environmental factors as potential pathogenic contributors to the disease is evident [3].

Cadmium, a widely exposed environmental hazard in air, soil, drinking water, and food, poses a substantial risk [4, 5]. Exposure pathways include smoking, soldering, and contact with cadmium-containing products such as nickel-chromium batteries, jewelry, toys, and electronic devices [6, 7]. However, in contrast to the widespread use of cadmium, there are concerns about the health risks associated with cadmium. Current research indicates that the accumulation of cadmium in the body can lead to hypertension, diabetes and cardiovascular disease [8, 9]. Besides, cadmium is also identified as a neurotoxic agent, may significantly contribute to the onset and progression of psychiatric disorders by influencing neurotransmitter release, oxidative stress responses, and inducing neuronal apoptotic procedures [10–12]. Previous research has linked cadmium to various psychiatric disorders, including bipolar disorder, schizophrenia, and major depression [11, 12]. Moreover, there is evidence suggesting that cadmium may elevate the risk of depression in the general population [13].

Smoking is a significant source of cadmium exposure in the United States. Previous research has demonstrated a strong correlation between smoking and depression, and this link may be bidirectional: smoking may increase the risk of depression, while depression may increase smoking behavior [14, 15]. Therefore, it is crucial to account for smoking-related variables when examining the association between cadmium and depression. Although smoking is more prevalent in men [16], research validates that women are more prone to depression [17–19]. However, limited research has explored the association between cadmium and depression in women, leaving the potential role of cadmium in the etiology of depression unclear. Therefore, it is imperative to conduct studies examining the correlation between blood cadmium levels and depression prevalence among females. This research utilized data from the National Health and Nutrition Examination Survey (NHANES) to investigate this relationship among adult females in the United States.

## 2. Methods

### Study population and design

The Centers for Disease Control and Prevention conducts a nationally representative survey known as NHANES. We acquired data from NHANES, a research initiative overseen by the National Center for Health Statistics (NCHS), designed to assess the health and nutritional status of the US population. The samples in the NHANES dataset demonstrate a high level of representativeness, primarily due to the meticulous implementation of a stratified multistage probability sampling methodology in the study design. The NCHS Research Ethics Review Board approved the inclusion of human subjects in the NHANES study, and all participants provided written informed consent. All NHANES data are publicly available at https://www.cdc.gov/nchs/nhanes/.

**Fig. 1:**
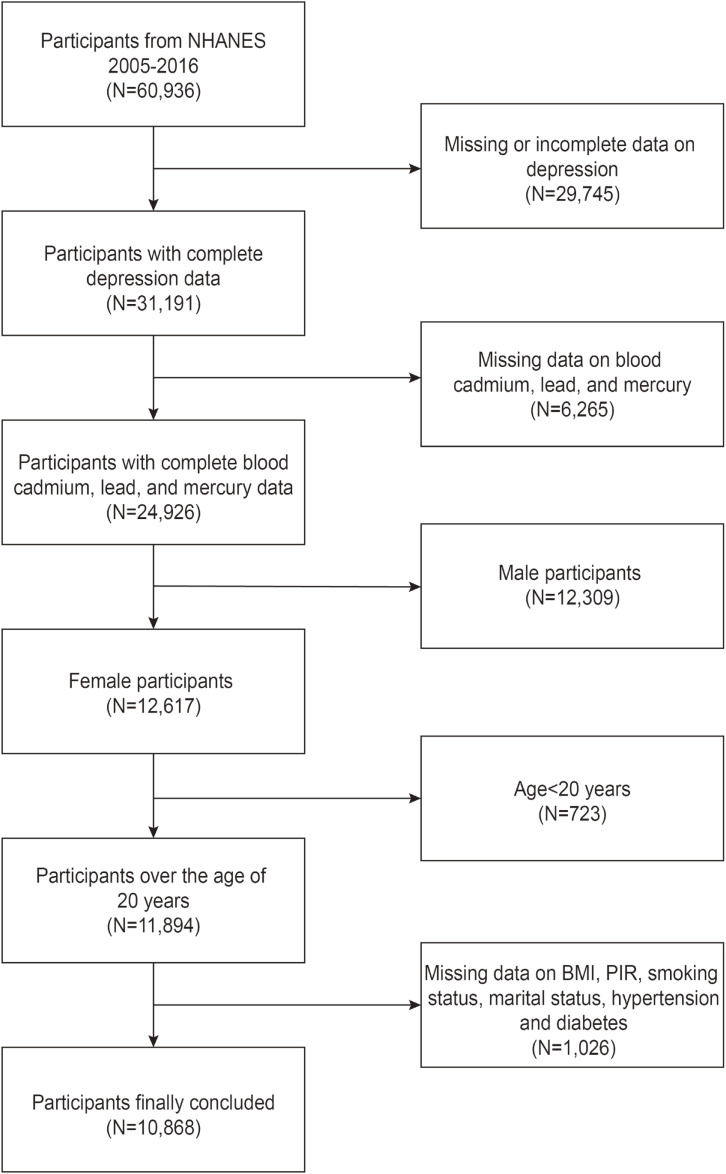
Flowchart of participants inclusion and exclusion.

Our study is grounded in data from six NHANES survey cycles spanning 2005 to 2016. Initially, 60,936 participants were recruited, and, following the exclusion of those with missing data on depression (n = 29,745), missing data on blood cadmium, lead and mercury concentrations (n = 6,265), males (n = 12,309), individuals younger than 20 years old (n = 723), and participants with missing data on other covariates (n = 1,026), a total of 10,868 participants were included in our study (Fig. 1).

### Exposure and outcome definition

Blood cadmium (BCd) was designed as the exposure variable. Blood cadmium is determined using inductively coupled plasma mass spectrometry. Detailed methodology and QA/QC instructions are discussed in the NHANES Laboratory Procedures Manual. (http://www.cdc.gov/nchs/data/nhanes/nhanes_07_08/PbCd_E_met_lead_cadmium.pdf; http://www.cdc.gov/NCHS/data/nhanes/nhanes_09_10/PbCd_F_met.pdf)

The NHANES employed the validated nine-item Public Health Questionnaire (PHQ-9) to measure depressive symptoms. The survey is well-known and offers a sensitivity and specificity of 88% [20]. The PHQ-9 is a highly effective tool for screening depressive symptoms, surpassing other screening tools in predictive value. Technical term abbreviations are explained when first used. It measures depressive symptoms in participants over the previous two weeks, including those experiencing restless sleep, loss of appetite, and loneliness. Each item is evaluated on a 4-point ordinal scale indicating the prevalence of the symptom (0, not at all; 1, several days; 2, more than half the days; 3, nearly every day). The PHQ-9 total score is the sum of the scores from the nine items. Therefore, participants with a score of 10 or higher were defined as having depressive symptoms. The prevalence of depression occurrence and the PHQ-9 score are designed as outcome variables in this study.

### Covariates

The covariates include age (years), gender (male/female), race (Mexican American/other Hispanic/non-Hispanic White/non-Hispanic Black/other races), body mass index (BMI), family income to poverty ratio (PIR), education level, smoking status (yes/no), marital status (yes/no), hypertension, and diabetes (Doctor informed you had any condition yes vs no). BMI was stratified into <25, 25–29.9, and ≥30 kg/m2, aligning with normal weight, overweight, and obesity classifications for all participants. PIR is a ratio of self-reported income to the local poverty level. Participants were considered as smokers if they had smoked ≥100 cigarettes in their life. The disease history was defined as self-reported physician diagnosis of hypertension and diabetes. On the NHANES website’s official page (https://www.cdc.gov/nchs/nhanes), you may find explanations for all variables.

### Statistical analysis

All statistical analyses followed the Centers for Disease Control and Prevention (CDC) guidelines and adjusted for the complex multistage cluster survey design during analysis.

Demographic characteristics of the subjects were assessed by using t-tests and chi-square tests. Associations between ln transform blood cadmium (in quartiles) and depression and PHQ-9 score in different models were investigated using logistic regression and multiple linear regression. Blood cadmium levels (µg/L) spanned 0.07–0.22, 0.23–0.35, 0.36–0.59, and 0.6–10.8 for quartiles 1–4, respectively. In Model 1, no covariates were adjusted. Model 2 included adjustments for age and race. Model 3 was adjusted for age, race, BMI, PIR, education level, marital status, smoking status, hypertension, and diabetes. Smoothed curve fitting was used to assess further the nonlinear association between ln transform blood cadmium and depression. The association between different ln transform blood cadmium categories and depression was investigated through subgroup analyses and interaction tests. All analyses were performed with R (version 4.3.1) or Empowerstats (version 2.0). Statistical significance is defined as a two-sided p-value < 0.05.

## 3. Results

### Baseline characteristics of participants

The baseline demographic characteristics of the enrolled participants are presented in Table 1. A total of 10,868 adult women were included in this study, with an average age of 48.78 ± 17.74 years. The mean blood cadmium, lead, mercury level was 0.54 ± 0.60, 1.32 ± 1.12, 1.44 ± 2.06, respectively. And the average PHQ-9 score was 3.70 ± 4.56. Participants with depression were more likely to be smokers (54.82%) and non-Hispanic whites (41.94%). In comparison to participants without depression, those with depression had higher BMI levels and an increased risk of hypertension and diabetes.

**Table 1:** Basic characteristics of participants. (N = 10,868)

**Characteristics**	**Total**	**Non-depression**	**Depression**	*P*-value*

	**N = 10,868**	**N = 9,695**	**N = 1,173**	
Age, (year)	48.78 ± 17.74	48.95 ± 17.98	47.37 ± 15.58	0.014
Race, (%)				<0.001
Mexican American	15.69	15.71	15.52	
Other Hispanic	9.44	8.88	14.07	
Non-Hispanic White	46.21	46.73	41.94	
Non-Hispanic Black	20.66	20.36	23.10	
Other Races	8.01	8.32	5.37	
Education level, (%)				<0.001
Less than high school	23.77	22.21	36.66	
High school or GED	21.83	21.77	22.25	
Above high school	54.41	56.02	41.09	
Smoking status, (%)				<0.001
Yes	37.15	35.01	54.82	
No	62.85	64.99	45.18	
Marital status, (%)				<0.001
Yes	47.52	49.15	34.10	
No	52.48	50.85	65.90	
Hypertension, (%)				<0.001
Yes	35.48	34.25	45.61	
No	64.52	65.75	54.39	
Diabetes, (%)				<0.001
Yes	11.48	10.59	18.84	
No	86.40	87.32	78.77	
Borderline	2.12	2.08	2.39	
BMI, (kg/m^2^)	29.57 ± 7.61	29.31 ± 7.41	31.73 ± 8.79	<0.001
PIR	2.48 ± 1.62	2.57 ± 1.62	1.66 ± 1.36	<0.001
Blood cadmium, (µg/L)	0.54 ± 0.60	0.52 ± 0.58	0.73 ± 0.75	<0.001
Blood lead, (µg/dL)	1.32 ± 1.12	1.31 ± 1.11	1.37 ± 1.18	0.063
Blood mercury, (µg/L)	1.44 ± 2.06	1.48 ± 2.06	1.11 ± 2.04	<0.001
PHQ-9 score	3.70 ± 4.56	2.42 ± 2.49	14.29 ± 3.93	<0.001

Mean ± SD for: continuous variables, (%) for categorical variables.

PIR, family income to poverty ratio; BMI, body mass index; PHQ, Patient Health Questionnaire.

BMI was categorized as <25, 25–29.9, and ≥30 kg/m^2^, which corresponded to normal weight, overweight, and obese population, respectively.

### Association between blood cadmium and depression

**Fig. 2:**
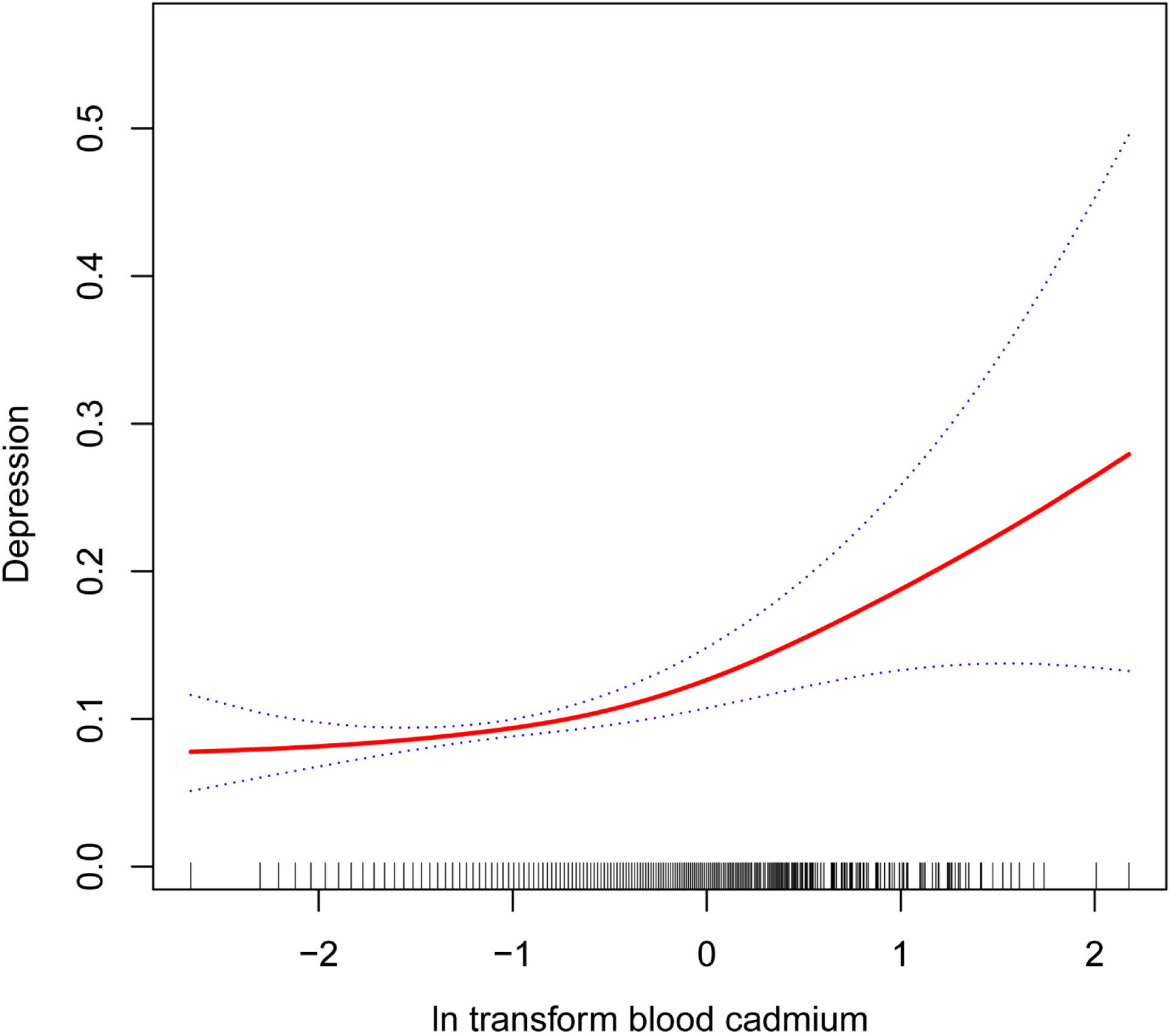
Non-linear correlation between blood cadmium and depression. The solid red line represent the smooth curve fit between variables. Blue bands represent the 95% of confidence interval from the fit.

Table 2 illustrates the relationship between ln transform blood cadmium levels and depression. The findings reveal a significant positive correlation between ln transform blood cadmium, depression, and PHQ-9 score. This positive association persists in the fully adjusted model (Model 3). A one-unit increase in ln transform blood cadmium is associated with a 33% higher depression prevalence (OR = 1.33, 95% CI: 1.21–1.45) and a 0.44 increase in PHQ-9 score (β = 0.44, 95% CI: 0.22–0.65). These associations maintain statistical significance after categorizing ln transform blood cadmium into quartiles (P for trend <0.05). Participants in the highest ln transform blood cadmium quartile face a 63% increased likelihood of depression (OR = 1.63, 95% CI: 1.15–2.30), with PHQ-9 score elevated by 0.73 (β = 0.73, 95% CI: 0.30–1.17). The association between other heavy metals such as blood lead, blood mercury and depression is shown in Table S1. Furthermore, the results from smoothed curve fitting affirm the nonlinear positive correlation between ln transform blood cadmium and depression (Fig. 2).

**Table 2:** Association between ln transform blood cadmium and PHQ-9 Score and Depression.

**ln transform blood cadmium level**	**PHQ-9 Score**	**Depression**

	**β (95% CI)**	**OR (95% CI)**
Crude model (Model 1)		
Continuous	0.71 (0.59, 0.82)	1.56 (1.45, 1.69)
Categories		
Quartile 1	0 (Ref)	1 (Ref)
Quartile 2	0.06 (−0.18, 0.30)	1.06 (0.87, 1.28)
Quartile 3	0.09 (−0.15, 0.33)	1.19 (0.98, 1.43)
Quartile 4	1.27 (1.02, 1.51)	2.19 (1.84, 2.60)
P for trend	<0.001	<0.001
Minimally adjusted model (Model2)		
Continuous	0.84 (0.73, 0.96)	1.67 (1.55, 1.81)
Categories		
Quartile 1	0 (Ref)	1 (Ref)
Quartile 2	0.20 (−0.05, 0.44)	1.13 (0.93, 1.38)
Quartile 3	0.35 (0.10, 0.60)	1.37 (1.12, 1.67)
Quartile 4	1.56 (1.31, 1.81)	2.61 (2.18, 3.12)
P for trend	<0.001	<0.001
Fully adjusted model (Model3)		
Continuous	0.44 (0.22, 0.65)	1.33 (1.21, 1.45)
Categories		
Quartile 1	0 (Ref)	1 (Ref)
Quartile 2	0.39 (0.03, 0.76)	1.19 (0.88, 1.61)
Quartile 3	0.17 (−0.21, 0.55)	1.10 (0.79, 1.52)
Quartile 4	0.73 (0.30, 1.17)	1.63 (1.15, 2.30)
P for trend	0.004	0.011

Model 1: no covariates were adjusted.

Model 2: age and race were adjusted.

Model 3: age, race, PIR, BMI, education level, smoking status, marital status, hypertension and diabetes were adjusted.

PIR, family income to poverty ratio; BMI, body mass index; PHQ, Patient Health Questionnaire.

### Subgroup analyses

In this study, we performed subgroup analyses and interaction tests by stratifying participants based on age, race, BMI, hypertension, diabetes, marital status, and smoking status to investigate the consistency of the relationship between ln transform blood cadmium and depression in the general population (Table 3). The findings reveal that this association shows no significant correlation with race, BMI, marital status, hypertension, and diabetes (P for interaction > 0.05). However, this positive association exhibited variations across age and smoking status subgroups. A one-unit increase in ln transform blood cadmium is linked to a 33% increase in the prevalence of depression among females under 60 years of age (OR = 1.33, 95% CI: 1.20–1.47). Conversely, this correlation lost significance in older females aged 60 and above (OR = 1.03, 95% CI: 0.83–1.27). Furthermore, the positive association remained significant in female participants who smoked (OR = 1.47, 95% CI: 1.31–1.64) but was not observed in the non-smoking population (OR = 0.98, 95% CI: 0.83–1.17). Our study’s outcomes suggest that the positive correlation between ln transform blood cadmium and depression remains consistent across different race groups, BMI categories, marital status, hypertension, and diabetes statuses, indicating its potential applicability in various population settings.

**Table 3:** Subgroup analysis of the association between ln transform blood cadmium and Depression.

**Subgroup**	**OR (95% CI)**	***P* for interaction**
Age		0.033
<60 years	1.33 (1.20, 1.47)	
≥60 years	1.03 (0.83, 1.27)	
Race		0.137
Mexican American	1.49 (1.14, 1.95)	
Other Hispanic	1.36 (1.04, 1.77)	
Non-Hispanic White	1.39 (1.22, 1.60)	
Non-Hispanic Black	1.20 (0.98, 1.47)	
Other Races	0.82 (0.53, 1.26)	
BMI		0.082
<25 kg/m^2^	1.39 (1.17, 1.66)	
25–29.9 kg/m^2^	1.47 (1.22, 1.77)	
≥30 kg/m^2^	1.17 (1.03, 1.33)	
Marital status		0.297
Yes	1.24 (1.07, 1.44)	
No	1.37 (1.22, 1.54)	
Hypertension		0.842
Yes	1.30 (1.14, 1.48)	
No	1.28 (1.15, 1.44)	
Diabetes		0.729
Yes	1.21 (0.95, 1.53)	
No	1.34 (1.21, 1.49)	
Borderline	1.32 (0.68, 2.56)	
Smoking status		<0.001
Yes	1.47 (1.31, 1.64)	
No	0.98 (0.83, 1.17)	

Age, race, BMI, marital status, smoking status, diabetes and hypertension were adjusted.

PIR, the ratio of family income to poverty; BMI, body mass index.

BMI was categorized as <25, 25–29.9, and ≥30 kg/m^2^, which corresponded to normal weight, overweight, and obese population, respectively.

## 4. Discussion

This cross-sectional investigation enrolled 10,868 adult women, revealing a notable discrepancy in blood cadmium levels between individuals with and without depression. Elevated blood cadmium levels correlated with an augmented risk of depression, a correlation persisting in the fully adjusted model. Furthermore, this positive correlation was consistent in subgroups stratified by race, BMI, marital status, hypertension, and diabetes statuses, suggesting the potential clinical utility of blood cadmium in depression assessment and prediction.

In recent years, more studies have reported a link between exposure to cadmium and mental health [19, 21, 22]. To our knowledge, this is the first study evaluating the connection between blood cadmium and depression in U.S. adult women. It is established that dysregulation of the monoaminergic neurotransmitter system may be implicated in depression onset. Cadmium, prevalent in daily life, infiltrates the body through avenues like smoking, welding, skin exposure, and contact with cadmium-containing products. It accumulates in the central nervous system, impacting neurotransmitter levels (serotonin, dopamine, and norepinephrine), potentially mediating depression onset [21, 23–25]. In addition, it has been suggested that heavy metal cadmium-mediated depression may be related to oxidative stress and neuroinflammatory mechanisms [21]. Animal experiments indicate that cadmium heightens the blood-brain barrier’s permeability, causing intracellular cadmium accumulation and impaired brain cell function [26, 27]. Rats exposed to cadmium displayed depression-like behavior in contrast to the control group, manifesting reduced neurotransmitter levels [27]. Evidence suggests cadmium exposure induces neurotoxic effects [28]. Additionally, elevated blood cadmium levels might elevate depression risk in South Korea’s elderly population [29]. Furthermore, an association between blood cadmium and depression was discerned in the 20–39 age group [7].

Smoking is a primary route of exposure to cadmium; tobacco plants absorb cadmium from the soil [30]. Heavy smokers, reportedly absorbing about one microgram of cadmium daily, exhibit the highest blood cadmium level [31]. Numerous studies affirm the link between smoking and depression, with smoking cessation lowering depression risk [32, 33]. Consequently, when investigating the cadmium-depression relationship, smoking is a crucial influencing factor, acknowledged as a significant covariate in our study.

Previous studies have shown that individuals with higher levels of blood cadmium have a higher incidence of depressive symptoms [7, 22] and are confounded by smoking factors [7]. Our study confirms this positive correlation in the general female population, significant in smokers and non-significant in non-smokers. A possible explanation may be that depressed people cope with their depression by smoking. It should be noted that the relationship between smoking and depression may be bidirectional: smoking may increase the risk of depression [14], and depression may increase the risk of smoking [15]. Our study identified variances in the link between blood cadmium and depression across different age subgroups. Specifically, we found a significant correlation among women under the age of 60 but not among older women over 60 years of age. This finding aligns with prior research that has focused on the connection between cadmium and depression among young adults aged between 20 to 39 years and 20 to 47 years [7, 34]. In summary, women’s blood cadmium levels may correlate with depression occurrence, although the underlying pathological mechanisms require comprehensive elucidation. Furthermore, a number of research indicates an association between other heavy metals in the blood, including blood lead and blood mercury, and depression [34, 35]. Lead exposure affects the catecholaminergic system of the central nervous system (CNS). Additionally, experimental animal studies have demonstrated that chronic exposure to lead results in reduced serotonin activity in the brain [36]. Mercury has been reported to cause psychiatric symptoms and disrupt serotonin metabolism in the brain, possibly by enhancing oxidative stress responses to the CNS [37]. However, consistent with previous studies [38, 39], this study did not find significant associations between blood lead and blood mercury concentrations and depression in US adult women.

This study possesses several strengths. Firstly, our research is based on NHANES data to enhance the representativeness of the study. Secondly, we adjusted for various confounding factors to ensure the study results’ stability and applicability to a broader population. Additionally, blood cadmium is considered a biomarker reflecting recent exposure, and our study is limited to participants’ depressive states recorded through PHQ-9 answers in the past two weeks, providing a better reflection of the current situation. Therefore, using blood cadmium as a recent exposure biomarker is appropriate in this context.

However, the study also has some limitations. Firstly, the questionnaire data in NHANES is subject to self-report limitations, and the covariates included in this study may not be entirely accurate, potentially affecting the precision of the results. In addition, due to the cross-sectional study design, this study could not determine the causal relationship between blood cadmium levels and depression, and our results should be interpreted with caution. Furthermore, this study was unable to investigate the potential association between cadmium exposure and chronic depressive symptoms over an extended period. Consequently, further large-scale prospective studies are needed to be designed to validate our findings.

## 5. Conclusion

In conclusion, our study revealed a positive association between higher blood cadmium levels and depression in adult women, but this association differed among subgroups of age and smoking status. Furthermore, the exact mechanistic pathways remain to be explored in depth.

## Abbreviations

BCd: Blood cadmium
CNS: Central nervous system
PHQ-9: Public Health Questionnaire
NHANES: National Health and Nutrition Examination Survey
NCHS: National Center for Health Statistics
BMI: Body mass index
PIR: Family income to poverty ratio

## Supplementary information

**Additional file 1:** **Table S1:** Association between other heavy metals lead and PHQ-9 Score and Depression.

## Declarations

### Data availability

Data used for this study are available on the NHANES website: https://wwwn.cdc.gov/nchs/nhanes/.
