# Supplementary material for: Association between blood cadmium and depression varies by age and smoking status in US adult women: a cross-sectional study from NHANES 2005–2016
Source: Environ Health Prev Med. 2024 Jun 22;29:32. doi: 10.1265/ehpm.24-00050 (PMC11211073; doi:10.1265/ehpm.24-00050)
Supplement: Supplementary file 1 — Additional file 1: Table S1: Association between other heavy metals lead and PHQ-9 Score and Depression. [file ehpm-29-032-s001.docx]

**Table S1:** Association between other heavy metals lead and PHQ-9 Score and Depression.

| **Exposure** | **PHQ-9 Score** | **Depression** |
| --- | --- | --- |
|  | **β (95% CI)** | **OR (95% CI)** |
| **ln transform blood lead level** |  |  |
| Crude model (Model 1) | 0.13 (0.00, 0.26) | 1.10 (1.01, 1.21) |
| Minimally adjusted model (Model 2) | 0.40 (0.25, 0.55) | 1.28 (1.15, 1.42) |
| Fully adjusted model (Model 3) | 0.23 (0.00, 0.46) | 1.16 (0.97, 1.39) |
|  |  |  |
| **ln transform blood mercury level** |  |  |
| Crude model (Model 1) | -0.47 (-0.56, -0.38) | 0.74 (0.69, 0.79) |
| Minimally adjusted model (Model 2) | -0.47 (-0.57, -0.38) | 0.73 (0.68, 0.78) |
| Fully adjusted model (Model 3) | -0.07 (-0.22, 0.07) | 0.97 (0.86, 1.10) |

Model 1: no covariates were adjusted.

Model 2: age and race were adjusted.

Model 3: age, race, PIR, BMI, education level, smoking status, marital status, hypertension and diabetes were adjusted.

PIR, family income to poverty ratio; BMI, body mass index; PHQ, Patient Health Questionnaire.
